# Comparison of Lethal and Nonlethal Mouse Models of *Orientia tsutsugamushi* Infection Reveals T-Cell Population-Associated Cytokine Signatures Correlated with Lethality and Protection

**DOI:** 10.3390/tropicalmed6030121

**Published:** 2021-07-02

**Authors:** Alison Luce-Fedrow, Suchismita Chattopadhyay, Teik-Chye Chan, Gregory Pearson, John B. Patton, Allen L. Richards

**Affiliations:** 1 Department of Viral and Rickettsial Diseases, Naval Medical Research Center, Silver Spring, MD 20910, USA; suchismita.chattopadhyay.ctr@mail.mil (S.C.); teikchye.chan.ctr@mail.mil (T.-C.C.); allen.richards@comcast.net (A.L.R.); 2 Department of Biology, Shippensburg University, Shippensburg, PA 17257, USA; gpearson@umass.edu; 3 Henry M. Jackson Foundation for the Advancement of Military Medicine, Bethesda, MD 20817, USA; 4 Department of Microbiology and Immunology, Thomas Jefferson University, Philadelphia, PA 19107, USA; john.patton@jefferson.edu; 5 Department of Preventive Medicine and Biostatistics, Uniformed Services University of the Health Sciences, Bethesda, MD 20814, USA

**Keywords:** scrub typhus, *Orientia tsutsugamushi*, mouse model, T cells, cytokines, lethality, protection

## Abstract

The antigenic diversity of *Orientia tsutsugamushi* as well as the interstrain difference(s) associated with virulence in mice impose the necessity to dissect the host immune response. In this study we compared the host response in lethal and non-lethal murine models of *O. tsutsugamushi* infection using the two strains, Karp (New Guinea) and Woods (Australia). The models included the lethal model: Karp intraperitoneal (IP) challenge; and the nonlethal models: Karp intradermal (ID), Woods IP, and Woods ID challenges. We monitored bacterial trafficking to the liver, lung, spleen, kidney, heart, and blood, and seroconversion during the 21-day challenge. Bacterial trafficking to all organs was observed in both the lethal and nonlethal models of infection, with significant increases in average bacterial loads observed in the livers and hearts of the lethal model. Multicolor flow cytometry was utilized to analyze the CD4+ and CD8+ T cell populations and their intracellular production of the cytokines IFNγ, TNF, and IL2 (single, double, and triple combinations) associated with both the lethal and nonlethal murine models of infection. The lethal model was defined by a cytokine signature of double- (IFNγ-IL2) and triple-producing (IL2-TNF-IFNγ) CD4+ T-cell populations; no multifunctional signature was identified in the CD8+ T-cell populations associated with the lethal model. In the nonlethal model, the cytokine signature was predominated by CD4+ and CD8+ T-cell populations associated with single (IL2) and/or double (IL2-TNF) populations of producers. The cytokine signatures associated with our lethal model will become depletion targets in future experiments; those signatures associated with our nonlethal model are hypothesized to be related to the protective nature of the nonlethal challenges.

## 1. Introduction

*Orientia tsutsugamushi*, an obligate, intracellular bacterium, is the causative agent of scrub typhus. Scrub typhus is a febrile disease, endemic to the Asia-Australia-Pacific region, with case fatality rates of up to 50% in untreated patients. In indigenous areas, scrub typhus can account for up to 19% of all illnesses and 23% of febrile illnesses [1]. The disease is transmitted to humans through the bite of infected chigger mites (*Leptotrombidium* spp.), and can lead to the production of an eschar at the bite site. Symptoms of the disease include fever, rash, pneumonitis, meningitis, and intravascular coagulation. The disease can be treated with doxycycline, tetracycline, or chloramphenicol. In vivo and in vitro, the bacteria can infect host cells that include macrophages, polymorphonuclear leukocytes, lymphocytes and endothelial cells [2,3,4]. Recent studies have shown the involvement of the NOD1-IL32 pathway in the production of inflammatory cytokines during *O. tsutsugamushi* infection [5]; the ability of *O. tsutsugamushi* to avoid autophagic defenses by host cells [6,7]; the necessity of IL1R signaling for host defense against *O. tsutsugamushi* and inflammasome activation in response to *O. tsutsugamushi* uptake [8]; the association of *O. tsutsugamushi* with lipid rafts during budding/exit from cells (but not during entry) [9]; and the upregulation of type 1 IFN genes, interferon-stimulated genes, and/or inflammatory cytokines/chemokines in infected human macrophages, characterized by a M1 polarization of macrophages [10]. Despite the advances in better defining the pathways/mechanisms that *O. tsutsugamushi* utilizes, no long lasting, broadly protective vaccine against scrub typhus has yet been developed [1]. Moreover, reports of scrub typhus not responding to appropriate antibiotic treatment are of concern [11]. Approximately one million cases of scrub typhus are reported each year in endemic areas, and this includes those cases reported among United States military personnel who are deployed/stationed overseas [12].

It is known that control of scrub typhus infections is under the regulation of both the cellular and humoral immune responses. The early immune response in mice includes macrophage- and dendritic cell-mediated cellular immunity, and is crucial to the clearance of infection [13,14]. Cell mediated immunity in mice has also been described in studies demonstrating protection from passive transfer of enriched spleen cells or T-cells [13,14,15,16,17,18,19], production of antigen specific lymphocytes [20,21,22], and delayed type hypersensitivity after immunization [16]. A humoral immune response during *O. tsutsugamushi* infections in mice has also been shown, e.g., antibody production from infection [15,23] and inhibition of establishment of infection in target cells by immune serum [24]. However, antibody production alone does not confer protection, as immunization of mice with irradiated *O. tsutsugamushi* protected against homologous/heterologous challenge, but minimal antibody response to the vaccination was observed [25].

Development of a successful vaccine will ultimately require a complete understanding of the host immune response, which includes detailing the profile(s) of immune cells that are elicited during *O. tsutsugamushi* infections. It has been suggested that the interactions of populations of immune cells such as T-cells, natural killer cells, dendritic cells, and macrophages, and how they relate to cytokine/chemokine production in vivo are a key component in the development of a scrub typhus vaccine [26,27] Studies aimed at defining the immune cell populations following infection with *O. tsutsugamushi* have been performed, particularly using human samples. One study performed flow cytometry analysis of patients’ immune cells during the acute and convalescent phases of disease to better define leukocyte populations [28]. Neutrophilia and CD4+ T lymphopenia (including T_reg_ cells) were observed during the acute phase of infection; increases in CD8+ cells were noted during the convalescent phase; and no changes in natural killer cells were noted [28]. Eschar skin biopsies from scrub typhus patients have also been analyzed using immunohistochemistry and microscopy tools to determine the phenotypes of infected host cells [28]. Within the eschar, *O. tsutsugamushi*-infected cells were found to be predominantly monocyte/macrophages and dermal dendritic cells, along with a smaller proportion of CD3+ T-cells. Further studies are needed to fully delineate the phenotypes of cells elicited during infection and their potential role(s) in the host immune response.

More than 20 antigenically distinct strains of *O. tsutsugamushi* have been reported to date [1,29]. The antigenic diversity of *O. tsutsugamushi* as well as the interstrain differences in virulence exhibited in mice enhance the necessity to dissect the host immune response. Murine susceptibility to *O. tsutsugamushi* infection and subsequent disease is dependent upon multiple variables, including challenge route, mouse strain, and *O. tsutsugamushi* strain. In particular, the intraperitoneal (IP) route of infection with the Karp strain of *O. tsutsugamushi* has been shown to consistently result in severe illness/death in a variety of inbred, outbred, and crossbred strains of mice [30]. Other strains of human pathogenic *O. tsutsugamushi* (e.g., Gilliam, Woods) administered by the IP route have shown various degrees of severe to moderate disease in the mouse model [30]. Intravenous (IV) models of infection in inbred mice have been shown to mimic the pathology of human scrub typhus cases [31,32,33] (IP models contrastingly result in peritonitis); however, IV models do not replicate the natural mode of infection (chigger to human). Intradermal models of infection, while mimicking chigger to human transmission, result in delayed bacterial trafficking and onset of clinical symptoms, and rarely cause lethality. In this study, the IP model was selected for lethal versus non-lethal analysis due to its reliability as a method that consistently provides both lethal (Karp strain) and non-lethal (Woods) infections. We hypothesized that distinct cytokine signatures within T-cell populations could be determined when comparing the lethal model (*O. tsutsugamushi* Karp intraperitoneal injection (Karp IP)) to nonlethal models (*O. tsutsugamushi* Karp intradermal injection (Karp ID), Woods intraperitoneal injection (Woods IP), and Woods ID) using multicolor flow cytometry to detect the prevalence of interferon gamma (IFNγ), interleukin 2 (IL2), and/or tumor necrosis factor alpha (TNF) producing T-cell populations. Interstrain (Karp IP to Woods IP or Karp IP to Woods ID) and intrastrain (Karp IP to Karp ID) model comparisons were made at distinct time points based upon the severity of illness in the mice: (1) Karp IP, 10 days—onset of severe illness; (2) Karp IP, 14 days—severe illness; (3) Karp ID, 10, 14, 21 days and Woods IP, ID, 10, 21 days—mild to no illness. The classification of cytokine signatures associated with both the lethal and nonlethal models of murine *O. tsutsugamushi* infection will aid in contributing to a better understanding of the various clinical signs and symptoms associated with scrub typhus infections and in the deduction of the immunopathological mechanisms related to lethality and protection from disease.

## 2. Materials and Methods

### 2.1. Mice and Orientia tsutsugamushi Infections

CD-1 Swiss outbred mice (female, 6–8 weeks of age) were purchased from Charles River Laboratories, Inc. (Wilmington, MA, USA). Mice were initially housed in animal biosafety level (ABSL)-2 laboratories and were transferred to an ABSL-3 laboratory for adaptation one week prior to challenge experiments. Mice were inoculated either intraperitoneally (IP) or intradermally (ID) with10^3^ MuID_50_ of *O. tsutsugamushi* strain Karp (Papua New Guinea) or Woods (Australia) as previously described [34]. For all ID inoculations, mice were anesthetized using isofluorane (inhalation administration). ID injections of 10^3^ MuID_50_ of *O. tsutsugamushi* were performed at the right ear dorsum at a single site (5 µL of pre-titrated liver-spleen homogenate) using a 0.3 mL insulin syringe (Becton Dickinson, NJ, USA). Negative control animals received an IP or ID inoculation of sterile PBS buffer. Following inoculation, the mice were observed for signs of systemic disease for 21 days. Mice (*n* = 3–8 per group/time point) were euthanized at 10, 14 (Karp IP only), and/or 21 days following infection. All animal experimentation was performed under the approval of the Institutional Animal Care and Use Committee at the Naval Medical Research Center, Silver Spring, MD (Protocol Number: 11-IDD-26).

### 2.2. O. tsutsugamushi qPCR Assay

*O. tsutsugamushi* was detected in the liver, lung, spleen, kidney, heart, and blood at each time point using a previously described qPCR assay for the *Orientia*-specific 47 kDa gene [35]. Templates were based on genomic DNA extracted from the aforementioned organs using the Bullet Blender Blue (Next Advance, Inc., Averill Park, NY, USA) and the DNeasy tissue kit (Qiagen, Valencia, CA, USA) according to the manufacturer’s protocols. Reactions were performed using the Platinum qPCR UDG Supermix kit (Life Technologies, Carlsbad, CA, USA) and all qPCR assays were performed in an ABI StepOne Plus thermocycler (Foster City, CA, USA). For normalization of *O. tsutsugamushi* copy numbers, a qPCR assay (Integrated DNA Technologies, Inc., Coralville, IA, USA) for the mouse glyceraldehyde 3-phosphate dehydrogenase (GAPDH) gene was used: forward primer 5′-AACTTTGGCATTGTGGAAGG-3′, reverse primer 5′-ACACATTGGGGGTAGGAACA-3′, and probe 5′-FAM-ACCCAGAAGACTGTGGATGG-3′. The relative *O. tsutsugamushi* genome levels were calculated after normalization to the GAPDH gene(s) using a standard curve that was constructed using 10-fold dilutions of known concentrations of an *O. tsutsugamushi* 47 kDa target sequence cloned into a plasmid, as previously described [36]. For negative controls, qPCR reactions of organs collected from uninfected mice and reactions containing no DNA template were included with each qPCR run. No copies of *O. tsutsugamushi* were detected in the negative controls.

### 2.3. ELISAs

Serum samples from mice at all time points were analyzed for the presence *O. tsutsugamushi*-specific antibodies (total IgG) as previously described [36]. Whole cell antigen (0.3 μg/100 μL; 0.1 μg from each strain of *O. tsutsugamushi* (Karp, Gilliam, and Woods) (diluted in sterile PBS) was passively adsorbed onto each well of one half of a 96-well plate (Dynatech Laboratories Inc., Chantilly, VA, USA). PBS (no antigen) was added to the other half of the 96-well plate. Coated plates were incubated at 4 °C in a refrigerator for at least 48 h, and then washed three times with 300 μL/well of wash buffer (PBS containing 0.1% Tween 20 (Sigma Chemical Co., St. Louis, MO, USA)). Plates were blocked with wash buffer containing 5% skim milk for 1 h at room temperature and then washed three times. Serum samples were serially diluted (four-fold dilutions from 1:100 to 1:6400) in wash buffer containing 5% skim milk to a final volume of 100 μL/well and incubated at room temperature for 1 h, and then were washed three times with wash buffer. The presence of specific antibodies was demonstrated by the subsequent binding of 100 μL/well of horseradish peroxidase-conjugated goat antibodies directed to mouse immunoglobulin G (IgG) (Kirkegaard and Perry Laboratories, Gaithersburg, MD, USA). The plates were incubated at room temperature for 1 h, washed, and 100 μL/well substrate (equal volumes of 2,2′-azino-di-(3-ethylbenzthiazoline-6-sulfonate) (ABTS) and hydrogen peroxide (Kirkegaard and Perry Laboratories)) were added to each well. The plates were incubated at room temperature for 15 min. Optical densities at 405 nm were measured by Vmax/Kinetic Microplate Reader (Molecular Devices, Sunnyvale, CA, USA). Titers were expressed as the inverse of the highest dilution in which a net optical density (absorbance with antigen minus absorbance without antigen) of 0.200 or greater was obtained. The mean net absorbance plus 3 standard deviations of three negative control sera was consistently less than an optical density of 0.200.

### 2.4. Flow Cytometry

At 10, 14 and 21 days following challenge, mice were euthanized and the spleens were processed into cell suspensions containing 1 × 10^7^ cells/mL in RPMI 1640 (Life Technologies) supplemented with 10% FBS and 50 µm BME. One million spleen cells were plated in a round bottom 96-well plate and stimulated with 1.5 µg of Kpr47 *O. tsutsugamushi* antigen (recombinant full-length 47 kDa protein from the Karp strain, produced by Invitrogen, Waltham, MA, USA) (experimental groups), 2 µg of ConA (positive control) or no stimulation (negative control) and were incubated for 2 h at 37 °C. Following incubation, 1 µL/mL of Golgi Plug (BD Biosciences, Franklin Lakes, NJ, USA) was added to the wells and the cells were incubated for 4 h at 37 °C and then transferred to 4 °C overnight. Cells were surface stained for 1 h at 4 °C with anti-mouse CD3 Alexa 700 (BD #557984), anti-mouse CD4 PerCP (BD #553052), and anti-mouse CD8 Pacific Blue (BD #558106). Cells were then permeabilized using the Cytofix/Cytoperm kit according to manufacturer’s instructions (BD #555029) and stained intracellularly for 2 h at 4 °C (in the dark) with anti-mouse IFNγ APC (BD #554413), anti-mouse IL2 FITC (BD #554427), and anti-mouse TNFα (BD #554419). Cells were washed in Perm/Wash Buffer (BD), resuspended in PBS, and acquired on an LSRII (BD Biosciences). Data were analyzed using FlowJo software version 7.6.5 (FLOJO LLC, Ashland, OR, USA).

### 2.5. Statistical Analysis

Comparisons between lethal and nonlethal experimental groups were performed using Students two-tailed *t*-test and/or Spearman’s Rank Correlation. A *p* value of ≤0.05 was considered significant. All statistical analyses were performed using Microsoft Excel 365 (2016), GraphPad Prism 7 (GraphPad Software, Inc., La Jolla, CA, USA), and/or R version 3.2.3.

## 3. Results

### 3.1. Clinical Observation and Morbidity/Mortality in Differentially Challenged Mice

**Figure 1:**
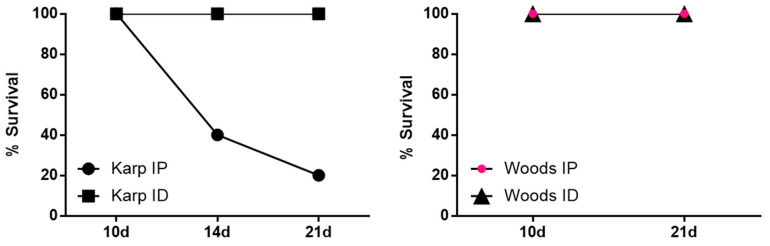
Comparison of survival in lethal and nonlethal murine models of *O. tsutsugamushi* infection, based upon strain and challenge route. All animals challenged with strain Woods (intraperitoneally (IP) or intradermally (ID)) and with strain Karp (ID) survived. Animals challenged with strain Karp (IP) demonstrated 40% and 20% survival at 14 dpi and 21 dpi, respectively.

The course of infection in mice challenged IP with *O. tsutsugamushi* Karp or Woods, or challenged ID with *O. tsutsugamushi* Karp or Woods was monitored for up to 21 days post infection (twice per day). No eschar formation and/or reaction at the injection site were observed in any of the differentially challenged mice, similar to previous studies [34,37]. Mice challenged IP with the Karp strain began to display signs of illness at 7 days post infection (dpi), including ruffled fur in 25% of the animals. By 9 dpi, 100% of the animals displayed ruffling of the fur and markedly slower movement. Mice became very ill by 12 dpi and displayed symptoms that included bloody noses, hunched posture, and difficulty in locomotion; very ill mice were euthanized in accordance with the approved IACUC protocol. At 14 dpi, 40% of mice challenged by Karp IP were alive and by 21 dpi, 20% of mice were alive (*n* = 1) (Figure 1). For those mice challenged ID with Karp, 100% survival was observed throughout the 21 day challenge experiment (Figure 1). The only outward symptom of illness displayed by the mice challenged ID with Karp was a slight ruffling of the fur beginning at 17 dpi in 40% of the animals and continuing in 100% of the animals from 19–21 dpi. Despite the ruffled fur, all Karp ID challenged animals were bright, alert, and reactive.

When mice were challenged with *O. tsutsugamushi* Woods, either IP or ID, 100% survival was observed during the 21 day challenge experiment (Figure 1). In the Woods IP challenged animals, we observed mildly ruffled fur in 100% of the animals at 17 dpi. At 18 dpi, the mildly ruffled fur was accompanied by abdominal bloating in 100% of the animals; these symptoms lasted until the end of the challenge experiment (21 dpi) (Table 1). In the ID challenged animals, abdominal bloating was observed in 100% of the animals from 19–21 dpi. Despite the observed symptoms in the IP and ID challenged animals, no behavioral changes were observed in any of the animals.

At the time of necropsy, visual inspections of the spleen, kidneys, liver, lungs, heart, and stomach were compared/recorded between challenged and control animals. In the IP Karp-challenged animals (*n* = 5), accumulation of bloody fluid in the peritoneum (100% of mice), enlarged spleens (2–3 times the size of control animals; 100% of mice), pale coloration of the liver (100% of mice), pale coloration of the kidneys (20% of mice), and pale coloration of the heart (20% of mice) were observed (Table 2). At 14 dpi, all IP Karp-challenged mice were very ill at the time of necropsy. In these animals, swelling of the stomach, enlarged spleen (1.5–2 times the size of control animals), pale coloration of the liver, and accumulation of bloody fluid in the peritoneum were observed (100% of animals) (Table 2). By 21 dpi, a single Karp-IP challenged mouse remained alive and displayed symptoms such as ruffled fur and decreased activity. During necropsy bloody fluid in the peritoneum, enlarged spleen (5–6 times the size of control mice), pale coloration of the kidneys, and enlargement of the liver and lungs were observed (Table 2). In the ID Karp-challenged mice at 10 dpi, enlarged spleens (2–3 times the size of control mice; 100% of mice) and a small amount of bloody fluid in the peritoneum (20% of mice) were observed (Table 1). At 21 dpi, the ID Karp-challenged mice displayed enlarged spleens (4–6 times the size of control mice; 100% of mice), enlargement of the liver and lungs (100% of mice), pale color of the kidneys (100% of mice), and a small amount of bloody fluid in the peritoneum (40% of mice) (Table 1).

Visual inspection of the organs in the Woods IP-challenged animals as compared to the control animals at 10 dpi revealed enlarged spleens (2–3 times the size of control animals; 71% of mice) and bloody fluid in the peritoneum (14% of mice) (Table 2); visual inspection of two of the mice did not reveal any differences in organs compared to control animals. At 21 dpi, the Woods IP-challenged animals showed enlarged spleens (4–5 times the size of control animals; 87.5% of mice) and an enlargement of stomach and small intestines (12.5% of mice) (Table 2); visual inspection of one of the mice did not reveal any differences in organs compared to control animals. In the Woods ID-challenged mice at 10 dpi, enlarged spleens were observed (2–3 times the size of control animals; 100% of mice) and a small amount of bloody fluid in the peritoneum (14% of mice) (Table 1). At 21 dpi, enlarged spleens were observed in all of the Woods ID-challenged mice (3–4 times the size of control mice; 100% of mice) (Table 1).

### 3.2. Determination of Antibody Titer in O. tsutsugamushi-Infected Mice

**Figure 2:**
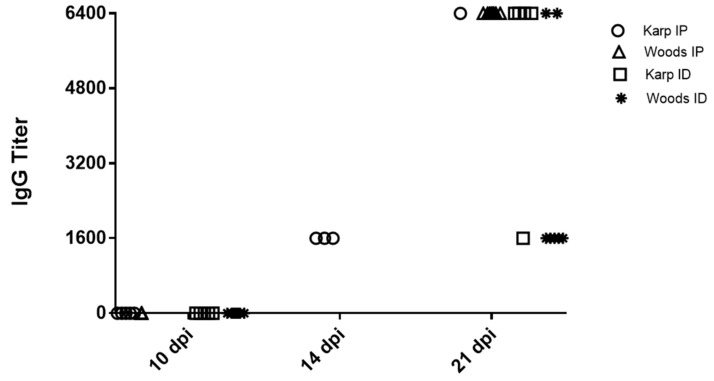
Detection of *O. tsutsugamushi*-specific IgG in serum of challenged mice at 10, 14, and 21 dpi. At 10 dpi, IgG was not detected in any of the challenged mice. At 14 dpi, 100% of Karp IP mice (*n* = 3) (only mice assessed at 14 dpi). At 21 dpi, 100% of the mice (all strains and challenge routes) (*n* = 5–8) displayed seroconversion. No *O. tsutsugamushi*-specific antibodies were detected in any of the negative control mice (data not shown).

The antibody response against *O. tsutsugamushi* (total IgG) was assayed at all time points (10, 14, and 21 dpi) in each mouse. No *O. tsutsugamushi*-specific antibodies were detected in any of the negative control mice. At 10 dpi, seroconversion was not detected in mice from any of the challenge groups (Karp IP, ID or Woods IP, ID) (Figure 2). At 14 dpi, 100% of mice (*n* = 3) in the Karp IP-challenge group displayed seroconversion (each with a titer of 1:1600) (Figure 2). At 21 dpi, 100% of mice in all challenge groups displayed seroconversion: the single surviving Karp-IP challenged mouse displayed a titer of 1:6400; in the Karp ID-challenge group (*n* = 5), 20% of mice displayed a titer of 1:1600 and 80% displayed titers of 1:6400; in the Woods IP-challenge group, 100% of mice (*n* = 8) displayed titers of 1:6400; and in the Woods ID-challenge group (*n* = 7), 71% of mice displayed titers of 1:1600 and 29% of mice displayed titers of 1:6400 (Figure 2).

### 3.3. Bacterial Trafficking of O. tsutsugamushi in Lethal and Nonlethal Models of O. tsutsugamushi Infection

**Figure 3:**
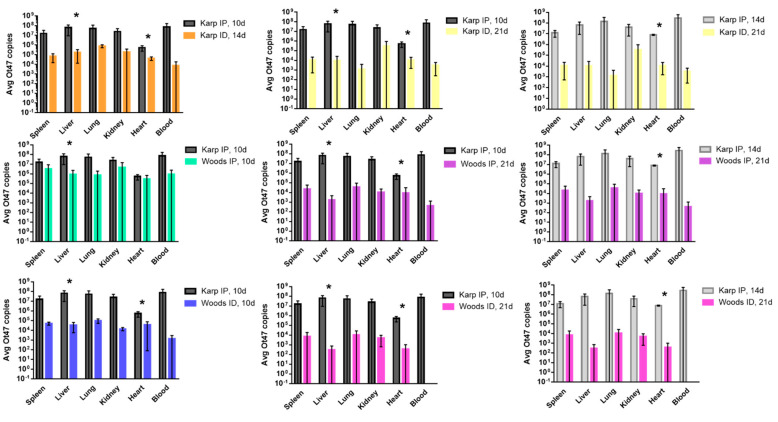
Bacterial trafficking of *O. tsutsugamushi* in the lethal (Karp IP) and nonlethal (Karp ID, Woods IP, Woods ID) models of *O. tsutsugamushi* infection at 10, 14, and/or 21 dpi. Significant differences between average bacterial loads in the lethal and nonlethal models are indicated by asterisks (*, *p* < 0.05).

A qPCR assay for the *Orientia*-specific 47 kDa gene [35] was used to assess the bacterial loads in spleen, liver, lung, kidney, heart, and blood samples collected from each mouse in the lethal (Karp IP) and nonlethal groups (Karp ID, Woods IP, and Woods ID) at each time point throughout the experiment (10, 14, and 21 dpi). Bacterial loads were measured in all organs in the lethal and nonlethal models at 10, 14, and 21 dpi; the lethal model (10 and 14 dpi) displayed increased bacterial loads in all organs compared to the nonlethal models (Figure 3). In the lethal model, the highest average bacterial loads were detected in the blood, liver, and lungs at both 10 dpi and 14 dpi. In the nonlethal models (Karp ID, Woods IP, and Woods ID), the highest average bacterial loads were detected in lungs or kidneys (Figure 3). Significant differences between the lethal and nonlethal models were only observed in bacterial loads found in the liver and/or the heart (Figure 3). Intrastrain comparisons revealed a significantly increased average bacterial load in the livers and hearts of the lethal model (Karp IP) at 10 dpi (start of severe illness) when compared to the nonlethal model (Karp ID) at 14 dpi or 21 dpi. By 14 dpi (severe illness) in the lethal model, a significant increase in the average bacterial load in the heart was detected when compared to mice in the nonlethal group at 21 dpi (Figure 3). Similar significant trends were observed during interstrain comparisons: (1) significantly increased average bacterial loads were observed in the livers and hearts of mice in the lethal group (Karp IP, 10 dpi) when compared to the nonlethal models Woods IP (21 dpi) and Woods ID (10 and 21 dpi); (2) significantly increased average bacterial loads were observed in the livers of mice in the lethal group (Karp IP, 10 dpi) when compared to the nonlethal model Woods IP (10 dpi); and (3) significantly increased average bacterial loads were observed in the hearts of mice in the lethal group (Karp IP, 14 dpi) when compared to the nonlethal models Woods IP (21 dpi) and Woods ID (21 dpi) (Figure 3).

### 3.4. Multifunctional Cytokine Analysis of O. tsutsugamushi-Specific CD4+ and CD8+ T Cells

**Figure 4:**
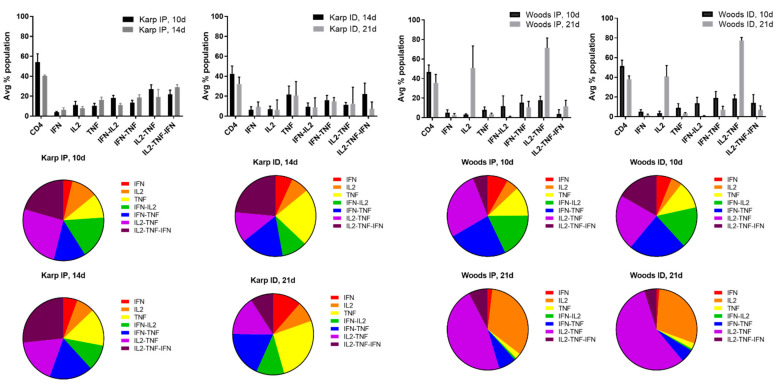
Multifunctional cytokine analysis of *O. tsutsugamushi*-specific CD4+ T cells represented by the frequency of cytokine (single, double, or triple) positive cells for the lethal (Karp IP) and nonlethal (Karp ID, Woods IP, and Woods ID) models of murine *O. tsutsugamushi* infection. The intracellular cytokine profiles for IFNγ, IL2, and TNF in individual cells were measured by multicolor flow cytometry with gating for CD4+ or CD8+ T cells. All possible combinations of cytokines are shown on the *x*-axis; the frequency of each population is shown as the mean ± SD on the *y*-axis.

**Figure 5:**
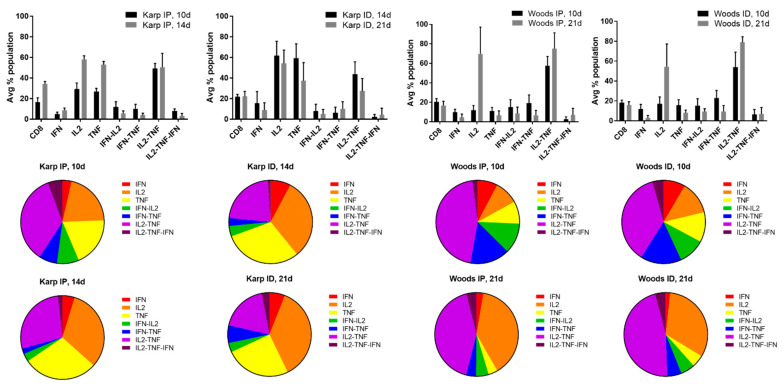
Multifunctional cytokine analysis of *O. tsutsugamushi*-specific CD8+ T cells represented by the frequency of cytokine (single, double, or triple) positive cells for the lethal (Karp IP) and nonlethal (Karp ID, Woods IP, and Woods ID) models of murine *O. tsutsugamushi* infection. The intracellular cytokine profiles for IFN, IL2, and TNF in individual cells were measured by multicolor flow cytometry with gating for CD4+ or CD8+ T cells. All possible combinations of cytokines are shown; the frequency of each population is shown as the mean.

In order to identify the cytokine signatures associated with the lethal or nonlethal model(s) of *O. tsutsugamushi* infection, multifunctional cytokine analysis of *O. tsutsugamushi*-specific CD4+ and CD8+ T cells was performed. Seven combinations of three cytokines (IFNγ, IL2, TNF, IFNγ-IL2, IFNγ-TNF, IL2-TNF, IL2-TNF-IFNγ) associated with CD4+ or CD8+ T-cell populations were compared between the lethal and nonlethal models (Figure 4 and Figure 5).

Cytokine production by individual cells of CD4+ T-cell populations in the lethal challenge groups was dominated by the triple production of IL2-TNF-IFNγ and the double production of IFNγ-IL2, IFNγ-TNF, and/or IL2-TNF at both 10 and 14 dpi; T-cell populations producing single cytokines comprised the smallest proportion of both of the lethal challenge groups (Figure 4). With one exception (Karp ID, 14 dpi), the dominance of triple producers was not observed in the nonlethal models (Karp ID, 21 dpi; Woods IP 10, 21 dpi; and Woods ID 10, 21 dpi) (Figure 4). Instead, double producers (IFNγ-TNF and/or IL2-TNF) tended to dominate the CD4 T-cell populations in the majority of the nonlethal groups (Karp ID, 21 dpi; Woods IP, 10, 21 dpi; Woods ID 10, 21 dpi) (Figure 4). Additionally in the nonlethal groups, single producers (IL2 and TNF) comprised a larger proportion of the CD4+ T-cell populations in several instances: Karp ID, 14 dpi (TNF); Karp ID, 21 dpi (TNF); Woods IP, 21 dpi (IL2); and Woods ID, 21 dpi (IL2) (Figure 4).

In distinct contrast, cytokine production by CD8+ T-cells in the lethal groups was not dominated by triple cytokine producing cells; in fact, the frequencies of the triple cytokine producing population were among the lowest in both of the lethal groups (Karp IP, 10 and 14 dpi) (Figure 5). Instead the lethal groups were dominated by CD8+ cytokine populations composed of primarily double (IL2-TNF) and single (IL2 and TNF) producers. Comparatively, in all instances but one (Woods IP, 10d), we also observed enhanced frequencies of single producers (IL2 and/or TNF); double producers (IL2-TNF) were also dominant among the CD8+ T-cell populations in all of the nonlethal groups (Figure 5).

The observed differences in dominance of triple, double, and single cytokine producing T-cell populations aligned with the hypothesis that distinct cytokine signatures could be associated with the lethal and nonlethal models of *O. tsutsugamushi.*

### 3.5. CD4+ and CD8+ T-Cell Cytokine Signatures of Lethality or Non-Lethality (Protection)

**Figure 6:**
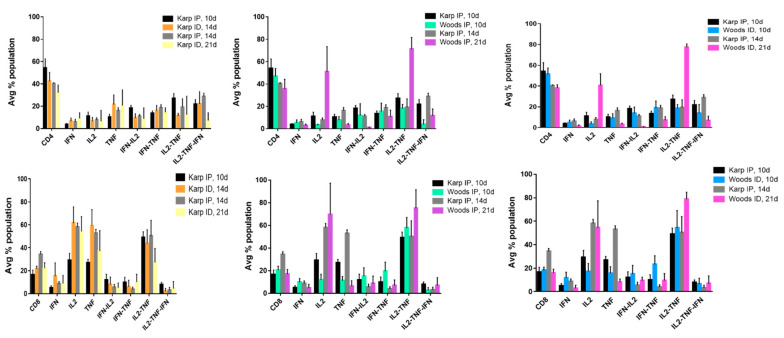
Multifunctional cytokine analysis of *O. tsutsugamushi*-specific CD4+ or CD8+ T cells represented by the frequency of cytokine (single, double, or triple) positive cells. Inter- and intrastrain comparisons are shown for the lethal (Karp IP) and nonlethal (Karp ID, Woods IP, and Woods ID) models of murine *O. tsutsugamushi* infection. The intracellular cytokine profiles for IFNγ, IL2, and TNFα in individual cells were measured by multicolor flow cytometry with gating for CD4+ or CD8+ T cells. All possible combinations of cytokines are shown on the *x*-axis; the frequency of each population is shown as the mean ± SD on the *y*-axis.

Cytokine signatures were determined by identifying the significant differences in CD4+ or CD8+ cytokine populations when comparing the lethal (Karp IP, 10 and 14 dpi) and nonlethal (Karp ID, 14 and 21 dpi; Woods IP, 10 and 21 dpi; Woods ID, 10 and 21 dpi) models of *O. tsutsugamushi* infection (Figure 6). Intra- and interstrain comparisons were made by comparing the lethal groups at the start of severe illness (10 dpi) or at a peak in severe illness (14 dpi) to each of the nonlethal groups (Figure 6). The observed significant differences in T-cell cytokine populations were defined as signatures of lethality or protection when they: (1) occurred in more than one experimental group of animals (lethal vs. nonlethal), thereby indicating a trend in production; and (2) demonstrated significant difference from the respective sham controls; i.e., those differences identified as significant in either the lethal or nonlethal groups also differed significantly from the sham controls.

**Figure 7:**
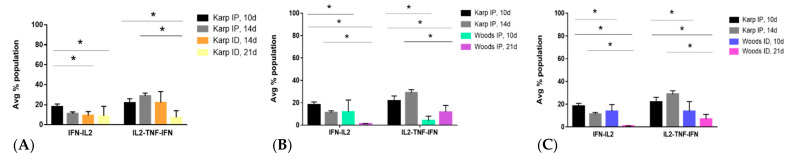
CD4 lethal cytokine signature. (**A**) Intrastrain comparisons revealed significant increase in IFN-IL2 production in the lethal model at 10 dpi compared to the nonlethal models at 14 and 21 dpi; significant increases in IL2-TNF-IFN production were observed in the lethal model at 10 and 14 dpi compared to the nonlethal model at 21 dpi. (**B**,**C**) In both interstrain comparisons, significant increases in IFN-IL2 and IL2-TNF-IFN were observed in the lethal model at 10 and 14 dpi when compared to nonlethal models at both 10 and 21 dpi. Significant differences between average cytokine populations in the lethal and nonlethal models are indicated by asterisks (*, *p* < 0.05).

When performing intra- and interstrain comparison of the CD4+ T-cell populations associated with the lethal and nonlethal models (Figure 6), significant differences in IFNγ-IL2 (double producers) and IL2-TNF-IFNγ (triple producers) cytokine populations were observed in the lethal model (Figure 7), thereby defining the lethal cytokine signature. Intrastrain comparisons revealed significant increase in IFNγ-IL2 production in the lethal model at 10 dpi compared to the nonlethal models at 14 and 21 dpi (Figure 7A). Intrastrain comparisons also revealed significant increases in IL2-TNF-IFNγ production in the lethal model at 10 and 14 dpi compared to the nonlethal model at 21 dpi (Figure 7A). In both interstrain comparisons, significant increases in IFNγ-IL2 and IL2-TNF-IFNγ were observed in the lethal model at 10 and 14 dpi when compared to nonlethal models at both 10 and 21 dpi (Figure 7B,C).

**Figure 8:**
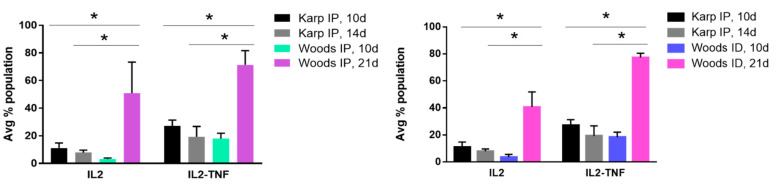
CD4 nonlethal (protective) cytokine signature. Significant differences in IL2 and IL2-TNF cytokine populations were observed in the interstrain comparisons between the lethal and nonlethal models. Significant increases in IL2 and IL2-TNF populations were observed in the nonlethal models at 21 dpi compared to the lethal models at both 10 and 14 dpi. Significant differences between average cytokine populations in the lethal and nonlethal models are indicated by asterisks (*, *p* < 0.05).

The CD4+ T-cell cytokine signature associated with the nonlethal infection model was defined by significant differences in IL2 and IL2-TNF cytokine populations observed in the interstrain comparisons between the lethal and nonlethal models (Figure 8). Significant increases in IL2 and IL2-TNF populations were observed in the nonlethal models (Woods IP and ID) at 21 dpi compared to the lethal model (Karp IP) at both 10 and 14 dpi. Significant nonlethal (protective) CD4+ T-cell cytokine signatures were not observed in the intrastrain comparisons of the lethal versus the nonlethal model.

**Figure 9:**
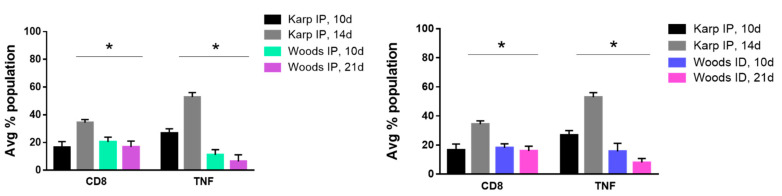
CD8 lethal cytokine signature. Significant differences in CD8 populations and TNF cytokine populations were observed in interstrain comparisons between the lethal and nonlethal models. In both interstrain comparisons, significant increases in CD8+ cells and TNF producing cells were observed in the lethal model at 14 dpi when compared to nonlethal models 21 dpi. Significant differences between average cytokine populations in the lethal and nonlethal models are indicated by asterisks (*, *p* < 0.05).

Significant differences between the CD8+ T-cell cytokine populations of the lethal and nonlethal groups were less numerous. A single producer (TNF) cytokine signature was observed in both of the interstrain comparisons; significant increases in TNF producing CD8+ T-cells were observed in the lethal model at 14 dpi when compared to the nonlethal models at 21 dpi (Figure 9). Concurrently, we also observed a significant increase in the overall frequency of CD8+ T-cells in the lethal model at 14 dpi (Figure 9). These significant increases were not observed in the lethal models at 10 dpi.

**Figure 10:**
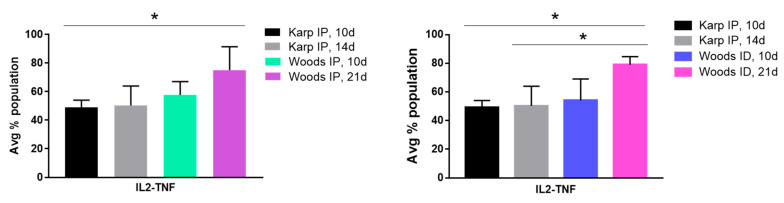
CD8 nonlethal (protective) cytokine signature. Significant differences in IL2-TNF cytokine populations were observed in the interstrain comparisons between the lethal and nonlethal models. A significant increase in the IL2-TNF population was observed in the nonlethal IP model at 21d compared to the lethal model at 10 dpi. A significant increase in the IL2-TNF population was observed in the nonlethal ID model at 21dpi compared to the lethal model at both 10 and 14 dpi. Significant differences between average cytokine populations in the lethal and nonlethal models are indicated by asterisks (*, *p* < 0.05).

Significant differences in the CD8+ IL2-TNF cytokine populations were observed in the interstrain comparisons; in particular, increases in IL2-TNF cytokine populations were observed in the nonlethal models (protective signature) in both interstrain comparisons (Figure 10). A significant increase in the IL2-TNF population was observed in the Woods IP nonlethal model at 21 dpi compared to the lethal model at 10 dpi (a slightly significant increase (*p* = 0.06) was also observed in the Woods IP nonlethal model at 21 dpi when compared to the lethal model at 14 dpi.). The significant increase in the CD8+ T-cell IL2-TNF population was also significantly increased in the Woods ID nonlethal model at 21 dpi compared to the lethal model at both 10 and 14 dpi (Figure 10).

### 3.6. Cytokine Signature Correlation to Bacterial Load

To determine if significant correlations existed between the identified signature CD4+ and CD8+ T-cell cytokine populations and splenic bacterial loads, Spearman’s correlation was used to analyze the potential relationship to disease activity. Correlation analysis was performed for all identified cytokine signatures, including: (1) CD4 lethal—IFNγ-IL2 and IL2-IFNγ-TNF; (2) CD4 nonlethal—IL2 and IL2-TNF; (3) CD8 lethal—TNF; and (4) CD8 nonlethal—IL2-TNF. No significant correlations between splenic bacterial load(s) and cytokine signatures were observed in any of the comparisons (data not shown).

## 4. Discussion

The purpose of this study was to identify the Th1-associated cytokine signatures of multifunctional T-cells (CD4+ or CD8+) in both lethal and nonlethal models of *O. tsutsugamushi*, in an effort to better define/refine the T-cell immune response associated with scrub typhus infections that range from mild to severe. Clinically, scrub typhus presents with flu-like symptoms, and in severe cases, pneumonia, renal failure, meningitis, gastrointestinal bleeding, and multi-organ failure can occur [38]. Because the early clinical presentation of scrub typhus is non-specific in nature and because of a lack of sensitive laboratory diagnostics for the first 5 days of disease presentation, misdiagnosis, delayed treatment, and/or inappropriate antibiotic administration can often be the cause for severe manifestations of the disease. Moreover, reports of scrub typhus failing to respond to antibiotic treatment have become more widespread in recent years [11,39]. The geographic distribution of *O. tsutsugamushi* comprises an area designated as the “Tsutsugamushi triangle”, which includes Pakistan [40], India [41], Nepal [42], Japan [43], Russia [44], Taiwan [45], China [46], Korea [47], Indonesia [48], Philippines [49], Thailand [50], and northern Australia [51]. Estimates suggest that *O. tsutsugamushi* causes one million clinical cases of scrub typhus per year and that approximately one billion people are at risk of contracting the disease [29]. Currently, reemergence of scrub typhus has been documented in regions of India, Nepal, Micronesia, and the Maldives [52]. More alarming, evidence of scrub typhus cases occurring in the Middle East [5], South America [53,54,55], and Africa [56,57,58] have recently been reported. The orientiae identified from United Arab Emirates [5] and Kenya [59] (*Candidatus* Orientia chuto), and from Chile (*Candidatus* Orientia chiloensis) [60] have been found to be related to, but significantly different from *O. tsutsugamushi*. Therefore, the emergence of scrub typhus cases poorly responsive to standard antibiotic treatment, large estimates of populations at risk for contraction of scrub typhus, the recently reported outbreaks, and the identification of the disease far from the Tsutsugamushi triangle all underscore the need for the development of new therapeutics, enhanced diagnostics, and ultimately a broadly protective vaccine.

To investigate host immune responses to scrub typhus that are associated with lethality and protection, we utilized a virulent strain (Karp) and a mildly virulent strain (Woods) of *O. tsutsugamushi* in a CD-1 outbred mouse model, challenged either intraperitoneally or intradermally, as previously described [34,61]. Karp IP challenge results in severe illness in mice beginning at 10 dpi, which becomes exacerbated by 14 dpi; therefore this challenge model and the associated time points were used to determine the cytokine signatures associated with lethal *O. tsutsugamushi* infection. By contrast, Karp ID, Woods IP, and Woods ID challenges result in milder observed infection in mice; hence the significant cytokine signatures associated with the Karp ID, Woods ID, and Woods IP models, when compared to the lethal models, were defined as signatures of nonlethality (protection). We monitored the physical signs of illness in all groups of mice, bacterial trafficking to multiple organs, and seroconversion in order to discern the course of infection associated with each challenge group. The severity of the course of infection was used as a parameter in order to delineate the challenge groups that would be used to determine the lethal and nonlethal (protective) T-cell cytokine signatures. In all challenge groups (Karp IP, ID and Woods IP, ID) we observed physical symptoms of illness, with the most severe and earliest onset occurring in the Karp IP challenge group; symptoms began at 7 dpi and were very severe by 12 dpi. In the nonlethal challenge groups, we also observed outward physical symptoms of illness (ruffling of fur and abdominal bloating); however these symptoms began later (17–19 dpi) than those observed in the lethal challenge group. Similarly, enhanced symptoms of organ involvement were observed in the lethal compared to the nonlethal challenge groups, including splenomegaly, fluid accumulation in the peritoneum, paling color of the liver, kidney, heart, and enlargement of the liver and lungs. Splenomegaly was observed in all of the nonlethal challenge groups, starting at 10 dpi and continuing to 21 dpi, and accumulation of fluid in the peritoneum was observed at 10 dpi. Bacterial trafficking to the spleen, liver, lung, kidney, and heart was also observed in both the lethal and nonlethal challenge groups throughout the course of the experiment. Average increased bacterial loads were observed in the lethal model at all time points compared to the nonlethal models, however the lungs in all challenge groups were among the organs containing the highest average bacterial loads, as has been previously reported for other murine models of *O. tsutsugamushi* infection [32,37,62,63]. Although average increased bacterial loads were observed in all organs in the lethal group, significant increases in average bacterial loads were only detected in the livers and/or hearts of the lethal challenge group. Seroconversion (total IgG) was detected in 100% of mice in all challenge groups by 21 dpi (titers of 1600 or 6400). Mice in the lethal challenge group (Karp IP) seroconverted earlier than all other challenge groups (14 dpi, titers of 1600). The combined observations of physical symptoms of infection, bacterial trafficking, and the humoral immune response allowed us to characterize the differential levels of severity associated with the lethal and nonlethal models of *O. tsutsugamushi*, as well as conclude that while the infection kinetics may differ between the two models, the elicited immune response and bacteremic levels are measurable and detectable throughout the course of both the lethal and nonlethal infection(s).

The observation of differential infection kinetics led us to hypothesize that distinct T-cell associated cytokine signatures could be prescribed to the lethal and nonlethal models of infection by identifying the significantly elevated T-cell associated cytokine populations in both models of infection. In particular, we were interested in determining if differential frequencies of multifunctional T-cells were elicited in the lethal and nonlethal challenge groups. Multifunctional T-cells (CD4+ and CD8+) have been implicated in both a protective (vaccine-induced and/or latent infections) as well as acute infection capacity during the immune response to various intracellular pathogens including *Mycobacterium tuberculosis* [64,65,66,67], *Francisella tularensis* [68], *Salmonella typhimurium* [69], *Leishmania* spp. [70,71], *Chlamydia* spp. [72] Varicella Zoster Virus [73], Human/Simian Immunodeficiency Virus [74], Hantaan Virus [75], and Ebola Virus [75]. Specifically, the frequencies of the singular as well as the simultaneous production of combinations of the Th1 cytokines IFNγ, IL2, and TNF-α have been attributed to immune correlates of protection or acute illness in the aforementioned studies. Accordingly, we examined the seven possible combinations of these three cytokines (IFNγ, IL2, TNF, IFNγ-IL2, IFNγ-TNF, IL2-TNF, IL2-TNF-IFNγ) in both the lethal and nonlethal models of *O. tsutsugamushi* infection, and determined which combinations served as signatures in each model.

In the lethal challenge group, we observed a dominance CD4+ T-cell populations composed of triple- and double-producing cells. Among these observed differences, significant increases in CD4+ T-cell populations producing IFNγ-IL2 and IL2-TNF-IFNγ were signatures of the lethal challenge group in both intra- and interstrain comparisons (Table 3). The significantly increased frequency of CD4+ T-cell populations that produced IFNγ-IL2 and IL2-TNF-IFNγ was detected starting at 10 dpi in the lethal group in both intra- and interstrain comparisons and remained significantly increased above all nonlethal challenge groups for the duration of the experiment (21 dpi). It has previously been demonstrated that the frequencies of these two specific populations of CD4+ multifunctional T-cells are correlated with active and/or latent disease status compared to uninfected and/or healthy controls. Triple-producing CD4+ T-cells have been correlated as markers of disease activity in cases of active tuberculosis [65,67,74,76], and CD4+ double producers of IFNγ-IL2 have also been implicated in increased/chronic disease activity and/or before administration of treatment(s) during active and latent tuberculosis infections [67,77,78]. T-cell differentiation has been hypothesized to occur in a linear fashion that is influenced by antigen exposure/stimulation, with progressive gains in functionality of the cells as they reach their peak of effector function optimization [79]. The generation of triple-producing CD4+ T-cells by continued antigenic stimulation was shown to lead to the generation of multifunctional central memory cells; however, continued antigenic stimulation was shown to be followed by a loss of memory potential as cells transitioned to a double production state (IFNγ-IL2) and ultimately to short-lived IFNγ-single-producing populations [79]. The finding that demonstrates the significant increase in CD4+ triple- and double-producing T-cells in the lethal *O. tsutsugamushi* model at the commencement and height of severe disease may suggest that a progressive loss of functionality of these populations is occurring as the infection progresses. Discrete detailing of the frequencies of triple, double, and ultimately single-producing (IFNγ) CD4+ T-cell populations at additional time points will be necessary for the full validation of this hypothesis. However, these data do suggest that elicitation of triple (IL2-TNF-IFNγ), double (IFNγ-IL2), and potentially single (IFNγ) CD4+ T-cell populations during *O. tsutsugamushi* vaccine candidate assessment may not offer the protective efficacy as previously described for other intracellular vaccine candidates [71,80,81]. As an additional consideration, the possibility exists that the enhanced production of these Th1 proinflammatory cytokine populations is contributing to the development of an aberrant cytokine storm leading to tissue destruction and consequent death in the host, as has been described for other mouse models of *O. tsutsugamushi* [82] and the closely related *R. conorii* [83].

In the nonlethal model, CD4+ T-cell triple-producing cytokine populations were outnumbered by a dominance of double-producing populations as well as single producers. Specifically, there was a significant increase in the frequency of CD4+ T-cell populations that produced IL2 and IL2-TNF in the nonlethal models at 21 dpi compared to the lethal models at 10 and 14 dpi during interstrain comparison (Table 3). It is well known that IL2 has an important role in the expansion of T-cell populations, has been correlated with T-cell functional quality, and is often a dominant cytokine during antigen clearance [64,84]. TNF-α has been shown to be a component of killing/control during *O. tsutsugamushi* [85,86,87] infections, and during infection with other intracellular pathogens [88,89,90,91,92], either alone or synergistically with IFNγ or IL2. The presence of multifunctional CD4+ T-cells that are double producers of IL2-TNF as well as the presence of IL2 single producing cells have been implicated in a protective capacity during vaccine challenge studies [71,80,93,94], during therapeutic treatment to prevent active infections [78,95], and in chronic versus acute infections [96] associated with intracellular pathogens. Vaccine studies related to intracellular pathogens have demonstrated that a significant elicitation of IL2 and IL2-TNF CD4+ T-cell populations are found in protected individuals and are closely related to the development/activation of memory T-cell populations (both central and effector) [71,80,93]; additionally, the presence of these cytokine populations has been correlated with the long term survival of primed T-cells [97,98]. IL2 has been hypothesized to promote the expansion of cells capable of producing TNF as an effector molecule [93], which aligns with the observed protective capacity observed with the significant increase in CD4+ T-cell populations producing IL2 and IL2-TNF in this study as well as other studies [93,99,100]. IL2 producing CD4+ T-cells have also been shown to express CD40L [101], promote the expansion of antigen specific B-cells, and/or supplement follicular Th cells to propel germinal center reactions [101,102,103]. Moreover, the level of antigenic stimulation can lead to exhaustion of T-cell populations; comparatively, low-level/slow antigen release can help to promote development of memory responses. In this study, the significant increase in IL2 and IL2-TNF was observed using the nonlethal intradermal challenge model. A commonality of the nonlethal ID model is the presence of lower levels of bacteria in the organs throughout the 21 day challenge experiment; therefore it can be hypothesized the intradermal challenge model allows for a slow release of antigen that better permits the development of CD4+ T-cell populations related to memory. The mechanisms associated with significant increases in the populations of CD4+ T-cell populations producing IL2 and IL2-TNF necessitate further investigation concerning their role during nonlethal *O. tsutsugamushi* infections. In particular, the development of central versus memory T-cell populations, the driving of antigen-specific B-cells, and the mode of challenge as potential mechanisms associated with protection deserve consideration from a pathogenic as well as a vaccine-development standpoint.

The role of multifunctional CD8+ T-cells and their associated signatures related to the lethal and nonlethal models of *O. tsutsugamushi* infections were less numerous; moreover, the overall numbers of CD8+ T-cells that were elicited in both the lethal and nonlethal models of infection were reduced in comparison to the CD4+ T-cell populations (Figure 4 and Figure 5). In the lethal model of infection, we did not observe increases in multifunctional CD8+ T-cell populations when compared to the nonlethal model. Instead we observed a significant increase in TNF producing populations (along with a concurrent increase in overall CD8+ T-cell frequency) in the lethal model at 14 dpi (severe illness) when compared to the nonlethal models at 21 dpi (interstrain comparisons only) (Table 4). It has been demonstrated that infection with *O. tsutsugamushi* elicits production of TNF (associated with both the acute and convalescent immune response) and increases in CD8+ T-cell populations [62,86,87,104]. Interestingly, we observed the same significant nonlethal signature associated with CD8+ T-cell populations as compared to CD4+ T-cell populations; IL2-TNF was found to be significantly increased in the Woods ID nonlethal model (interstrain comparison) at 21 dpi compared to the lethal model at 10 and 14 dpi, and in the Woods IP nonlethal model at 21 dpi when compared to the lethal model at 10 dpi (a slightly significant increase (*p* = 0.06) was observed at 14 dpi). It has recently been demonstrated in an inbred model of *O. tsutsugamushi* Karp infection (combined ID and subcutaneous (SQ) challenge) that CD8+ T-cells contribute to protection against lethal challenge [62]. Notably, the authors demonstrated that CD8+ T-cells were required to limit bacterial proliferation during the third week of challenge, that adoptive transfer of splenic CD8+ T-cells from ID/SQ-challenged mice could protect IP-challenged mice, and that expanded populations of CD8+ T-cells were needed to prevent recurrent growth of *O. tsutsugamushi* during the latent phase of infection [62]. The data from this study combined with the current observations of significant increases in IL2-TNF producing CD4+ and CD8+ populations in the nonlethal models of infection at 21 dpi potentially suggests that the production of IL2-TNF by splenic T-cells may mediate protection via IL2 expansion of TNF effector cells that may contribute to limiting/controlling bacterial replication and/or enhance the development of central and effector memory T-cell populations that contribute to the prevention of recurrent growth of *O. tsutsugamushi.* Expanded experimentation defining the associated effector functions and memory phenotypes of multifunctional T-cells will be imperative to concluding the distinct roles and multifunctional potential of both CD4+ and CD8+ populations; these characteristics will be useful in the future development of both vaccines and therapeutics.

In summary, we have shown that multifunctional CD4+ and CD8+ T-cells are elicited in both the lethal and nonlethal outbred murine models of *O. tsutsugamushi* infection. Multifunctional CD4+ and CD8+ T-cells were observed at the beginning and at the height of severe illness in the lethal model; in the nonlethal models, the multifunctional T-cells were observed throughout the course of the study (21 days). By comparing the lethal and nonlethal models, we were able to identify significant cytokine signatures associated with either lethality or protection. Mechanistic validation of the signatures will be necessary in order to determine if the identified populations of multifunctional T-cells are involved in events such as progressive T-cell differentiation and its relation to function, promotion of the expansion of antigen-specific B-cell populations, and/or the appropriate development of memory T-cell populations. As a mechanistic correlate of protection, the development of multifunctional T-cell populations may serve as a parameter of consideration in the formulation of scrub typhus vaccines. Thus, the cytokine signatures identified in this study may provide a starting point for evaluation of multifunctional T-cell populations in additional animal models, as well as in human samples. To fully understand the possible protective capacity of multifunctional T-cells (from a vaccine standpoint), additional functions/phenotypic characters (recognition of infected cells, differentiation trends, tissue-specific populations, persistence/memory development, preferential cytokine production based upon *Orientia* strain and route of challenge) will require investigation. Moreover, the populations/roles of innate immune cells as they correlate with multifunctional T-cell function in response to lethal and nonlethal challenge, as well as the multifunctional T-cell populations associated with sublethal *O. tsutsugamushi* challenge will be essential to an all-inclusive understanding of the murine immune response during scrub typhus infections; these experiments are currently underway in our laboratory.

## Figures and Tables

**Table 1:** Visual observations of affected organs observed in *O. tsutsugamushi* ID Karp- or Woods-challenged mice (compared to control animals) at the time of necropsy (10 and 21 dpi). The number of mice with affected organs (numerator) out of the total mice in the group (denominator) is indicated for each specific observation.

	10 dpi		21 dpi	
	Karp ID	Woods ID	Karp ID	Woods ID
Enlarged spleen	5/5	7/7	5/5	7/7
Fluid in peritoneum	1/5	1/7	2/5	
Pale liver				
Pale kidney			5/5	
Pale heart				
Enlarged stomach				
Enlarged liver			5/5	
Enlarged lungs			5/5	

**Table 2:** Visual observations of affected organs observed in *O. tsutsugamushi* IP Karp- or Woods-challenged mice (compared to control animals) at the time of necropsy (10, 14, and 21 dpi). The number of mice with affected organs (numerator) out of the total mice in the group (denominator) is indicated for each specific observation.

	10 dpi		14 dpi	21 dpi	
	Karp IP	Woods IP	Karp IP	Karp IP	Woods IP
Enlarged spleen	5/5	5/7	3/3		7/8
Fluid in peritoneum	5/5	1/7	3/3	1/1	
Pale liver	5/5		3/3		
Pale kidney	1/5			1/1	
Pale heart	1/5				
Enlarged stomach			3/3		1/8
Enlarged liver				1/1	
Enlarged lungs				1/1	

**Table 3:** Summary of the CD4+ T-cell population lethal and nonlethal (protective) cytokine signatures. Comparisons between lethal (Karp IP) and nonlethal (Karp ID, Woods IP, Woods ID) challenge models were performed in an intra- and interstrain fashion. Lethal signatures are designated by an asterisk (*) and nonlethal (protective signatures) are indicated by a hashtag (#), each indicating a significant increase (*p* < 0.05) in the associated population of cytokine-producing cells.

**Karp IP vs Karp ID**	**IFN**	**IL2**	**TNF**	**IFN-IL2**	**IFN-TNF**	**IL2-TNF**	**IL2-TNF-IFN**
10d vs 14d				*			
10d vs 21d				*			*
14d vs 21d							*
**Karp IP vs Woods IP**	**IFN**	**IL2**	**TNF**	**IFN-IL2**	**IFN-TNF**	**IL2-TNF**	**IL2-TNF-IFN**
10d vs 10d				*			*
10d vs 21d		#		*		#	*
14d vs 21d		#		*		#	*
**Karp IP vs Woods ID**	**IFN**	**IL2**	**TNF**	**IFN-IL2**	**IFN-TNF**	**IL2-TNF**	**IL2-TNF-IFN**
10d vs 10d				*			*
10d vs 21d		#		*		#	*
14d vs 21d		#		*		#	*

**Table 4:** Summary of the CD8+ T-cell population lethal and nonlethal (protective) cytokine signatures. Comparisons between lethal (Karp IP) and nonlethal (Karp ID, Woods IP, Woods ID) challenge models were performed in an intra- and interstrain fashion. Lethal signatures are designated by an asterisk (*) and nonlethal (protective signatures) are indicated by a hashtag (#), each indicating a significant increase (*p* < 0.05) in the associated population of cytokine-producing cells.

**Karp IP vs Karp ID**	**IFN**	**IL2**	**TNF**	**IFN-IL2**	**IFN-TNF**	**IL2-TNF**	**IL2-TNF-IFN**
10d vs 14d							
10d vs 21d							
14d vs 21d							
**Karp IP vs Woods IP**	**IFN**	**IL2**	**TNF**	**IFN-IL2**	**IFN-TNF**	**IL2-TNF**	**IL2-TNF-IFN**
10d vs 10d							
10d vs 21d						#	
14d vs 21d			*				
**Karp IP vs Woods ID**	**IFN**	**IL2**	**TNF**	**IFN-IL2**	**IFN-TNF**	**IL2-TNF**	**IL2-TNF-IFN**
10d vs 10d							
10d vs 21d						#	
14d vs 21d			*			#	

## Data Availability

Not applicable.

## Institutional Animal Care and Use Committee (IACUC) Statement

All animal experimentation was performed according to the guidelines and under the approval of the Institutional Animal Care and Use Committee (IACUC) at the Naval Medical Research Center, Silver Spring, MD, USA (Protocol Number: 11-IDD-26).
